# Transperitoneal Versus Extraperitoneal Approach for Laparoscopic and Robot-Assisted Radical Prostatectomy: A Systematic Review and Meta-Analysis

**DOI:** 10.5152/tud.2023.23008

**Published:** 2023-09-01

**Authors:** Stefanus Purnomo, Agus Rizal Ardy Hariandy Hamid, Moammar Andar Roemare Siregar, Andika Afriansyah, Hendy Mirza, Doddy Hami Seno, Nugroho Purnomo

**Affiliations:** 1 Department of Urology, Universitas Indonesia – Dr. Cipto Mangunkusumo Hospital, Faculty of Medicine, Jakarta, Indonesia; 2 Department of Surgery, Persahabatan General Hospital - Universitas Indonesia, Faculty of Medicine, Division of Urology, Jakarta, Indonesia

**Keywords:** Transperitoneal, extraperitoneal, laparoscopy, robot-assisted, radical prostatectomy

## Abstract

To conduct a comparative analysis of outcomes from 2 different surgical approaches, transperitoneal radical prostatectomy (TP-RP) and extraperitoneal radical prostatectomy (EP-RP) in minimally invasive surgery. A comprehensive search was conducted up to September 2022 using 5 online databases, namely PubMed, Cochrane, Scopus, EMBASE, and Science Direct. Studies were screened per the eligibility criteria, and outcomes included operative duration, estimated blood loss (EBL), hospital stay, operative complication, and positive surgical margin. Total of 13 studies compiled of 2387 patients were selected, with TP-RP and EP-RP performed on 1117 (46.79%) and 1270 (53.21%) patients, respectively. Six laparoscopy radical prostatectomy (LRP) studies and 7 robot-assisted radical prostatectomy (RARP) studies with 1140 and 1247 patients, respectively, were also included. The EP-RP demonstrated a marked advantage in terms of operative complications (Risk Ratio [RR] = 0.78, 95% CI = 0.62, 0.98; *P = *.04), but no significant difference concluded for operative duration, EBL, hospital stay, and surgical margin. In the RARP group, there was a significant difference in operative duration for EP-RARP and TP-RARP (Mean difference [MD] = −17.27, 95% CI = −26.89, −7.65; *P = *.0004), hospital stay (MD = −0.54, 95% CI = −0.94, −0.14; *P = *.008), and operative complications (RR = 0.7, 95% CI = 0.49, 0.99; *P = *.04). There were no noteworthy variations identified in EBL and surgical margin. Furthermore, the LRP group did not show any significant differences. This study shows that regardless of the techniques used, EP-RP has a lower risk of operative complications than TP-RP, with no significant difference in other outcomes.

## Introduction

The treatment of choice for localized prostate cancer is radical prostatectomy (RP), which is considered to be the most effective surgical approach.^1^ In comparison to the extraperitoneal radical prostatectomy (EP-RP) approach, the transperitoneal radical prostatectomy (TP-RP) approach is the most commonly used method, despite having contact with bowels and some advantages such as greater working area and quicker trocar preparation and positioning from the expanded workspace.^2^

Laparoscopy radical prostatectomy (LRP) is regarded as the conventional therapy for confined prostate cancer in numerous countries. The approach is still being debated and is dependent on surgeon preference. The transperitoneal laparoscopy radical prostatectomy (TP-LRP) approach creates larger working space and better visualization; however, extraperitoneal laparoscopy radical prostatectomy (EP-LRP) approach is preferred because there is no intraperitoneal organ involvement.^3,4^

The utilization of robot-assisted radical prostatectomy (RARP) is considered an outstanding surgical method used to treat prostate cancer that is localized. More than a decade ago, extraperitoneal RARP (EP-RARP) became one of the favored surgical approaches and was applied more frequently than transperitoneal RARP (TP-RARP). Based on a review by Wang et al,^5^ a subgroup meta-analysis of 13 studies revealed regardless of the higher rate of postoperative complications with TP-LRP, it has no statistical significance in the most important indicators compared to EP-LRP.

The fact that these 2 approaches are mostly considered for RP, yet the best approach is still being debated. Consequently, a systematic review and meta-analysis was carried out to evaluate the statistical significance from each approach to compare their operative outcomes and early postoperative period. Specifically, this study main points are to analyze the results of TP-RP and EP-RP in Radical Prostatectomy, to analyze them in LRP subgroup and last to analyze them in RARP subgroup.

## Material and Methods

To ensure this study was conducted in accordance with best practices, we used Preferred Reporting Items for Systematic Reviews and Meta-Analyses (PRISMA) guidelines.^6,7^ The study protocol has been registered in the International Platform of Registered Systematic Review and Meta-Analysis Protocols (INPLASY) database INPLASY2022110042. In addition, the selected studies were identified using the PICO (Population, Intervention, Comparison, and Outcome; Table 1) approach. The authors did not conduct any new human or animal experiments for this article and relied solely on previously published research; therefore, we did not require ethics committee approval and informed consent for this research.

### Search Strategy and Study Selection

To conduct a comprehensive search, an extensive exploration was conducted on 5 databases, namely PubMed, Cochrane, Scopus, EMBASE, and Science Direct, with the search period extending up to September 2022. The search terms employed are as follows “radical prostatectomy,” “transperitoneal,” “extraperitoneal,” “laparoscopy,” and “robot-assisted.” Only human studies published within the last 20 years were considered for selection. Additionally, the references of all studies that met the inclusion criteria were thoroughly examined, and if there were any differences from the screening, reviewers conducted a discussion. This research exclusively examines full-text studies that compare clinical outcomes in men who have undergone RP via either transperitoneal or extraperitoneal laparoscopy or robot-assisted laparoscopy, as shown in Supplementary Table 1.

### Study Eligibility

This review exclusively included studies that met specific inclusion criteria, focusing on patients who had undergone RP using laparoscopy or robot-assisted laparoscopy. The criteria required that the studies must make a comparison between the transperitoneal and extraperitoneal approaches and must be original research articles reporting outcomes such as estimated blood loss (EBL), surgical complications, operative duration, hospital stay, and positive surgical margin (PSM). Studies that were non-comparative, lacked full-text availability, did not separately report outcomes, or were published before 2002 were excluded from the analysis. 

### Screening

Two independent reviewers were responsible for the selection and inclusion of studies in this review (S.P. & M.A.R.S.). Duplicate studies were eliminated using EndNote X9 and screened based on titles/abstracts. The eligibility of full-text papers was then examined, and studies that matched were added to this study. 

### Data Extraction and Validity Assessment

All studies included in the review provided demographic and outcome data, including clinical variables such as age, body mass index (BMI), prostate-specific antigen (PSA), Gleason score, and Tumour, Node, Metastasis (TNM) staging. Perioperative variables such as operative duration, EBL, hospital stay, complications, and PSM were also documented. The collected data were separated into subgroup analytical studies. For studies that reported median values, the validated mean was utilized, while an estimated standard deviation was applied in studies lacking standard deviation data. To assess the potential for bias, The Cochrane Risk of Bias tool was used for randomized control trials (RCTs), and for cohort studies, the Newcastle–Ottawa Scale tool was used to assess bias in 3 areas: study selection, comparability, and outcome. Studies with scores of 7 or higher are considered good quality with low bias risk.

### Statistical Analysis

The selected studies for this meta-analysis were analyzed using The Cochrane Collaboration Review Manager (RevMan, version 5.4, The Cochrane Collaboration, 2020). Dichotomous variables were analyzed using risk ratio (RR) with a 95% confidence interval (CI), while continuous variables were calculated using mean difference (MD) with 95% CI. The outcomes evaluated for continuous variables were operative duration, EBL, and hospital stay, while operative complications and PSM were classified as dichotomous variable outcomes. *P *value less than .05 was termed as statistically significant. *I* ^2^ serves as a measure of heterogeneity and ranges from 0% to 100%. When *I* ^2^ is less than 25%, it indicates low heterogeneity. A value of 50% suggests moderate heterogeneity, and 75% suggests high heterogeneity. Depending on the level of heterogeneity, either the fixed effect model or the random effect model was used to calculate the pooled effect size. The fixed effect model was used when there was low heterogeneity, while the random effects model was used when there was significant heterogeneity.^8^

## Results

**Figure 1.**
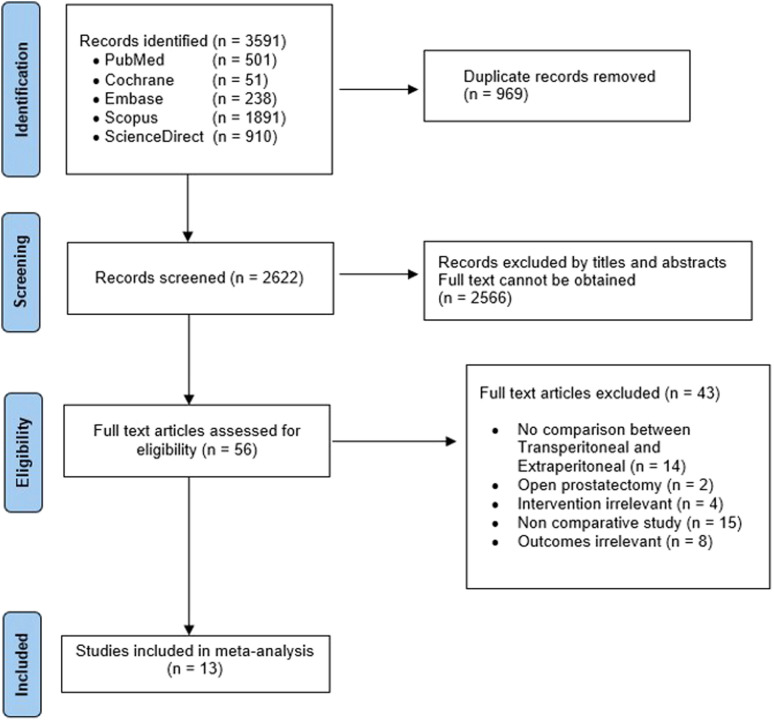
Preferred Reporting Items for Systematic Reviews and Meta-Analyses flow diagram.

The initial search results from all databases yielded 3591 studies, with all duplicates removed using the EndNote X9 application. Following duplicate removal and titles or abstracts screening, 56 studies were evaluated for eligibility. Consequently, there were 13 studies in the meta-analysis after exclusion, as shown in Figure 1. 

### Study Characteristics

**Figure 2.**
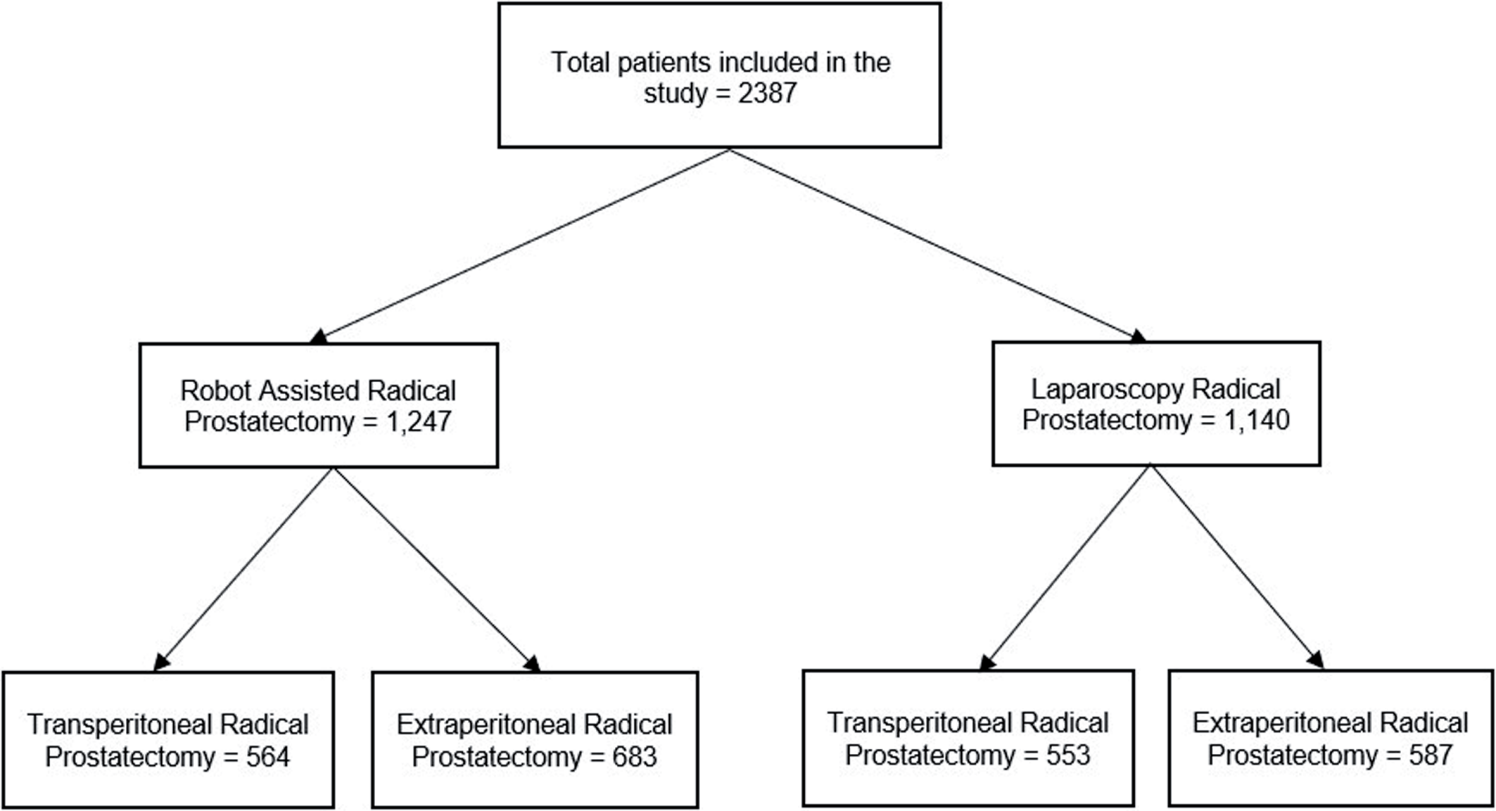
Patients included in the study.

Out of the 13 studies considered, 2 were RCT, 1 was a prospective cohort study, and the remaining 10 were retrospective cohort studies. There were 2387 patients in the meta-analysis, with TP-RP and EP-RP performed on 1117 patients (46.79%) and 1270 patients (53.21%), respectively. The LRP was carried out in 6 studies on 1140 patients (TP-RP group = 553 patients; EP-RP group = 587 patients), while 7 studies used RARP on 1247 patients (TP-RP group = 564 patients; EP-RP group = 683 patients) as shown in Figure 2. Studies originated from worldwide, such as the United States of America, Brazil, the United Kingdom, Switzerland, Turkey, India, China, Thailand, France, and Italy. Furthermore, the mean age, BMI, and PSA concentration for TP-RP were 64.14 years, 25.79 kg/m^2^, and 11.32 ng/dL, respectively, and 63.36 years, 25.79 kg/m^2^, and 11.89 ng/dL, respectively, for EP-RP. The meta-analysis included studies that compare transperitoneal and extraperitoneal approaches in laparoscopy or robot-assisted laparoscopy techniques, as shown in Table 2. 

### Assessment of Study Quality

The Cochrane Risk of Bias tool was used to evaluate all RCTs, and low bias was observed. For cohort studies, the Newcastle–Ottawa Scale was utilized to assess the risk of bias. The Newcastle–Ottawa Scale evaluated 3 factors to determine the risk of bias. Subsequently, 3 studies resulted in total score of 5 stars, 5 studies resulted in total score of 6 stars, 2 studies resulted in total score of 7 stars, and only 1 study resulted in score of 8 stars, as shown in Supplementary Figure 1.

### Operative Duration

**Figure 3.**
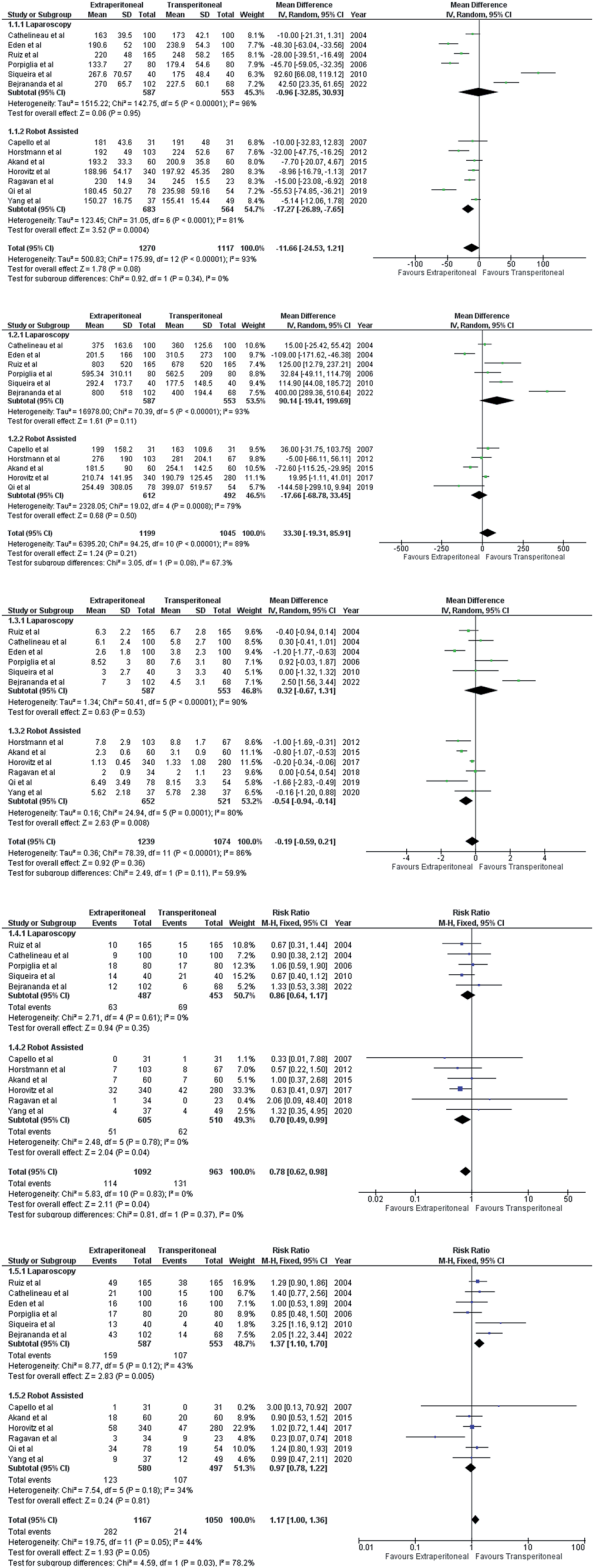
Meta-analysis of outcomes between extraperitoneal and transperitoneal. (A) Operative duration, (B) estimated blood loss, (C) hospital stay, (D) operative complication, (E) positive surgical margin.

This review encompassed a total of 13 studies, with 1270 patients undergoing EP-RP and 1117 patients undergoing TP-RP. The results between TP and EP did not yield any significant results with all approaches (MD = −11.66; 95% CI: −24.53, 1.21; *P* = .08; see Figure 3A). Similar outcome was found between TP-RP and EP-RP in the laparoscopy group (MD = −0.96; 95% CI: −32.85, 30.93; *P* = .95). However, in the robot-assisted group, comparing TP-RP and EP-RP, the EP approach was favored (MD = −17.27; 95% CI: −26.89, −7.65; *P* = .0004).

### Estimated Blood Loss

A total of 11 studies with 1199 EP-RP patients and 1045 TP-RP patients were included. The overall analysis between the 2 approaches did not reveal any significant difference for this particular outcome (MD = 33.30; 95% CI: −19.31, 85.91; *P* = .21; Figure 3B). There was no statistical significance between the 2 approaches used in the laparoscopy group (MD = 90.14; 95% CI: −19.41, 199.69; *P* = .11). Similar results were seen in the robot-assisted group, with no significant difference between the TP-RP and EP-RP approach (MD = −17.66; 95% CI: −68.78, 33.45; *P *= .50). 

### Hospital Stay

The meta-analysis included 12 studies, with 1239 patients undergoing EP-RP and 1074 patients undergoing TP-RP. The overall meta-analysis for these approaches did not reveal any significant difference between the TP-RP and EP-RP (MD = −0.19; 95% CI: −0.59, 0.21; *P* = .36; please refer to Figure 3C). In the laparoscopy group, no significant difference was observed between TP-RP and EP-RP approaches (MD = 0.32; 95% CI: −0.67, 1.31; *P* = .53). However, a statistically significant outcome was observed in the robot-assisted group between TP-RP and EP-RP favoring EP for a shorter hospital stay needed (MD = −0.54; 95% CI: −0.94, −0.14; *P* = .008). 

### Operative Complication

A total of 11 studies comparing operative complications with 1092 EP-RP patients and 963 TP-RP patients were included. Overall analysis revealed that EP approach was favored regarding operative complications (RR = 0.78; 95% CI: 0.62, 0.98; *P* = .04; please refer to Figure 3D). In the laparoscopy group, no significant difference was observed between the TP-RP and EP-RP approaches (RR = 0.86; 95% CI: 0.64, 1.17; *P* = .35). In contrast, a notable distinction was observed between TP-RP and EP-RP approaches in the robot-assisted group thus EP-RP produced lower risk complications (RR = 0.70; 95% CI: 0.49, 0.99; *P* = .04).

### Positive Surgical Margin

A total of 12 studies comparing TP-RP and EP-RP approaches were included, with 1167 EP-RP patients and 1050 TP-RP patients. In this outcome, the overall analysis showed no notable dissimilarity (RR = 1.17; 95% CI: 1.00, 1.36; *P* = .05; Figure 3E). The laparoscopy group showed that the TP approach has 37% more risk to PSM than EP approach (RR = 1.37; 95% CI: 1.10, 1.70; *P *= .005). However, the robot-assisted group did not show any significant difference (RR = 0.97; 95% CI: 0.78, 1.22; *P *= .81).

## Discussion

There are different options for RP; however, the majority of surgeons prefer the TP route over the EP route. The reason for this could be attributed to the surgeon's familiarity with the surroundings and the availability of more space for work, but it is still uncertain which approach is the best.^9^ Proficiency and knowledge are important factors in deciding the optimal approach; however, the availability of resources should also be taken into account. The RARP has become an important choice in the modern era due to its unique features that are considered advantages. Many developed countries have adopted RARP due to some of the advantages of using advanced technology in surgical procedures including the availability of flexible operation equipment, better vision, ease of learning, better ergonomics, reduced hand tremors, and improved dexterity.^10-13^ Although RARP has been found to have better outcomes in terms of post-surgery results such as PSM, urine continence, and sexual function in systematic reviews and meta-analyses, certain studies have pointed out that the cost of RARP may be higher than LRP because of the expensive surgical instruments utilized.^14,15^ The LRP approach is now widely used in most developing nations to treat localized prostate cancer. The EP and TP surgeries remain technically challenging. While TP-LRP is frequently used, EP-LRP has benefits such as avoiding bowel contact and a quicker return to a regular diet.^16^

Although there is evidence suggesting that the EP approach leads to a slightly shorter operative time, this difference of 11.66 minutes did not reach the typical threshold for statistical significance.The 2 approaches in the LRP group had no statistical significance, while the EP-RARP outperformed the TP-RARP in the RARP group, with a significant difference of shorter operative time of 17.27 minutes. Similarly, Uy et al^19^ obtained a significant difference in operative duration, with total operation time being shorter in EP-RARP than in TP-RARP (MD: −14.4 min; 95% CI: −26.3, −2.4: *P = *.02). This was due to adding a shorter duration for trocar insertion to compensate for the prolonged console time since there was no exposure to the bowels in EP-RARP. The EBL results in this study revealed no significant difference (MD: 33.30; *P = *.21). There are benefits and drawbacks to both approaches. While TP is more common, EP has certain benefits such as no contact with the bowel, allowing for a quicker return to normal food intake and lower risk of damage to organs within the abdominal cavity. On the other hand, the EP-RP approach has some disadvantages, such as a narrower surgical field and limited visual access. Furthermore, the EP technique had a longer operation time from skin incision to skin suture due to space creation.^17,18^ Similar results were found for hospital stay with no statistical significance (MD: −0.19; *P* = .36). Nonetheless, a substantial distinction was observed in the RARP category for EP-RARP and TP-RARP with shorter hospital stay favoring the EP approach. These factors contributed to faster discharge time which correlated with no bowel contact, resulting in less nausea, faster oral intake by patients, and less time required to recover postoperatively.

Based on operative complications, the EP-RP was associated with a 22% risk reduction of operative complications compared to the TP approach. The outcome was not statistically significant in the LRP group, while it was statistically significant in the RARP group favoring the EP approach with 30% less risk of complications compared to TP. Furthermore, the EP approach is superior due to the absence of exposure to bowels. The EP-RP approach avoids injury to the bowel and reduces the risk of complications such as hernias and ileus by not incising the transversalis fascia and peritoneum. Similar complication rates with more complications after TP were reported in all cited studies.^19-21^ In contrast, other studies found no statistical significance with a similar number of complications occurring in both approaches and no patients having severe complications (Clavien-Dindo class III-V).^22,23^ In the landscape of surgical techniques, the EP and TP methods diverge, highlighting differences in instrument interactions, pelvic lymph node dissection, and postoperative bowel function. The EP approach involves surgery outside the peritoneal cavity, utilizing small abdominal incisions to minimize instrument conflicts with intra-abdominal organs. In contrast, the TP approach enters the peritoneal cavity, potentially leading to instrument clashes among abdominal organs.^24^ The EP approach often allows for targeted pelvic lymph node dissection, while the TP approach offers comprehensive lymph node removal, particularly valuable for aggressive cancer treatment. In terms of postoperative recovery, the EP approach generally supports quicker bowel function restoration due to its less invasive nature, whereas the TP approach, involving peritoneal manipulation, might entail a transient delay. These complications include ileus, urinary and fecal incontinence, urine retention or leaking, bleeding of the urethra, and erectile dysfunction, with the greatest concern for urologists being erectile dysfunction and urinary incontinence. Minor complications could still occur and tend to resolve completely after appropriate treatment in each case.^25-27^ Research from Kallidonis et al^28^ resulted overall complications TP revealed more complications compared to EP approach starting from ileus, urine leakage, acute urinary retention, urinary tract infections, and bladder neck stenosis with significant number. While on EP approach, lymphocele seems to occur more than on TP. In terms of early continence rates, the EP group exhibited a favorable outcome, with statistical significance observed only within the laparoscopic subgroup (Odd Ratio (OR) 2.52, *P* < .001). However, no statistically significant difference emerged between the EP and TP cohorts regarding 12-month continence rates in both the laparoscopic (OR 1.55, *P* = .12) and robotic subgroups (OR 3.03, *P* = .21).^28 ^The statistical analysis in this study did not reveal any significant difference between the TP and EP groups in terms of PSM (RR: 1.16; *P = *.22). The subgroup analysis did not find any statistically significant difference between the LRP and RARP groups, which was consistent with other studies that evaluated the incidence of PSMs. This implies that the findings of this study are consistent with previous studies.^29-31^

The limitation of this study includes the fact that some of the retrospective studies were of medium quality, and only 3 studies conducted were of high quality, despite the low risk on 2 RCT studies. However, the advantage was that high heterogeneity levels between studies were seen in the outcomes of each group. Heterogeneity was expected due to the inclusion of various international studies from around the world, which were combined with some studies with missing outcome parameters, resulting in the exclusion of outcome analyses. Further studies evaluating RP methods should be conducted with more complex outcomes analyses in each approach to produce more specific results.

## Conclusion 

This study revealed a thorough comparison between TP-RP and EP-RP outcomes and the LRP and RARP methods for each approach. Regardless of the techniques used, the outcomes of interest clearly revealed that EP-RP had a lower risk of operative complications than TP-RP. Furthermore, the results of other parameter outcomes were similar, with no significant difference between TP-RP and EP-RP. Our results enhance the existing body of evidence and have the potential to improve the practice recommendations of professional societies.

## Figures and Tables

**Table 1.** Population, Intervention, Comparison, and Outcome Approach

Population	Men with a history of radical prostatectomy
Intervention	Extraperitoneal radical prostatectomy
Comparison	Transperitoneal radical prostatectomy
Outcome	Operative duration Estimated blood loss Hospital stay Surgical complication Positive surgical margin

**Table 2.** Characteristics of Included Studies

Study	Study Design	Country	Group	Approach	Sample Size	Age (Year)	BMI (kg/m^2^)	PSA (ng/dL)	Gleason Score	TNM Stage
Cathelineau et al 2004	Cohort	France	LRP	TP	100	63	25	8.9	NA	T1: 68%, T2: 32%, T3: 0%
				EP	100	61	26	10	NA	T1: 72%, T2: 27%, T3: 1%
Eden et al 2004	Cohort	United Kingdom	LRP	TP	100	62.3	NA	7.7	≤8: 100%	T1: 64%, T2: 36%, T3: 0%
				EP	100	61.4	NA	7.6	≤8: 100%	T1: 48%, T2: 48%, T3: 4%
Ruiz et al 2004	Cohort	France	LRP	TP	165	64.1	NA	10.8	2-4: 20% 5-6: 57.6% 7: 17% 8-9: 5.4%	T1a-b: 4.8% T1c: 66.7% T2a: 25.5% T2b: 3%
				EP	165	62.9	NA	9.9	2-4: 10.3% 5-6: 50.9% 7: 32.2% 8-9: 6.7%	T1a-b: 3% T1c: 64.2% T2a: 30.3% T2b: 2.4%
Porpiglia et al 2006	Cohort	Italy	LRP	TP	80	64.25	24.5	8.35	≤7: 82.5% >7: 1.25%	T1c: 72.5% T2: 27.5%
				EP	80	64.4	24.8	9.7	≤7: 86.2% >7: 6.25%	T1c: 65% T2: 35%
Capello et al 2007	RCT	United States of America	RARP	TP	31	59	26.5	6.1	NA	T2a: 13%, T2b: 6.45%, T2c: 58%, T3a:19.35%, T3b: 3.2%
				EP	31	56	29.8	29.8	NA	T2a: 29%, T2b: 9.7%, T2c: 51.7%, T3a: 3.2%, T3b: 6.4%
Siqueira et al 2010	Cohort	Brazil	LRP	TP	40	59.8	NA	5.4	4 (2 + 2): 0% 5 (3 + 2): 0% 6 (3 + 3): 50% 7 (3 + 4): 35% 7 (4 + 3): 15%	T1c: 80% T2a: 17.5% T2b: 2.5%
				EP	40	63.6	NA	5.9	4 (2 + 2): 2.5% 5 (3 + 2): 5% 6 (3 + 3): 80% 7 (3 + 4): 5% 7 (4 + 3): 7.5%	T1c: 50% T2a: 37.5% T2b: 12.5%
Horstmann et al 2012	Cohort	Switzerland	RARP	TP	67	65.6	26.3	12.2	<6: 62% 7: 36% >8: 2%	T1: 18% T2: 64% T3: 18%
				EP	103	64.4	26.5	7	<6: 62% 7: 36% >8: 2%	T1: 50% T2: 43% T3: 7%
Akand et al 2015	RCT	Turkey	RARP	TP	60	60.5	27.4	8.6	≤6: 55% 7: 38.3% ≥8: 6.7%	T1b: 5%, T1c: 80%, T2a: 11.7%, T2b: 3.3%, T2c: 0%, T3a: 0%
				EP	60	60.8	26.1	9	≤6: 68.3% 7: 31.7% ≥8: 0%	T1b: 0%, T1c: 60%, T2a: 26.7%, T2b: 8.3%, T2c: 1.7%, T3a: 3.3%
Horovitz et al 2017	Cohort	United States of America	RARP	TP	280	62.32	29.65	6.91	5 & 6: 31.8% 7: 54.6% 8: 10.7% 9 & 10: 2.9%	T1a: 0.36% T1c: 67.5% T2a: 20.36% T2b: 7.86% T2c: 3.57% T3a: 0.36%
				EP	340	61.04	28.98	5.95	5 & 6: 66.2% 7: 30% 8: 3.2% 9 & 10: 0.6%	T1a: 0% T1c: 77.94% T2a: 17.94% T2b: 1.47% T2c: 2.65% T3a: 0%
Ragavan et al 2018	Cohort	India	RARP	TP	23	66	25.7	15	6: 47.8% 7: 43.5% 8: 8.7% 9: 0%	Benign: 8.7% T2: 52.2% T3: 39.1%
				EP	34	65.5	24.2	10.63	6: 38.3% 7: 41.2% 8: 14.7% 9: 5.8%	Benign: 0% T2: 70.6% T3: 29.4%
Qi et al 2019	Cohort	China	RARP	TP	54	70.5	23.98	24.51	≤6: 3.7% 7: 48.1% >7: 48.1%	T1-T2: 70.4% T3-T4: 29.6%
				EP	78	66.77	24.19	24.17	≤6: 14.1% 7: 52.6% >7: 33.3%	T1-T2: 80.8% T3-T4: 19.2%
Yang et al 2020	Cohort	China	RARP	TP	49	67.43	24.4	18.06	NA	T1: 0% N0: 50% T2: 40.8% N1: 50% T3: 59.2% M0: 100% M1b: 0%
				EP	37	67.4	24.99	12.26	NA	T1: 2.7% N0: 71.4% T2: 56.8% N1: 28.6% T3: 40.5% M0: 97.3% M1b: 2.7%
Bejrananda et al 2022	Cohort	Thailand	LRP	TP	68	69.1	24.5	14.7	6: 23.5% 7: 58.9% 8: 7.4% 9: 10.3%	T1b: 2.9% T1c: 2.9% T2a: 5.9% T2b: 7.4% T2c: 44.1% T3a: 16.2% T3b: 20.6% T4: 0%
				EP	102	68.5	24.2	12.7	6: 34.4% 7: 49% 8: 7.8% 9: 8.8%	T1b: 0% T1c: 0% T2a: 8.8% T2b: 5.9% T2c: 54.9% T3a: 6.9% T3b: 22.5% T4: 1%

All data represented as mean, except Gleason score and TNM stage.

BMI, body mass index; EP, extraperitoneal; LRP, laparoscopy radical prostatectomy; PSA, prostate-specific antigen; RARP, robot-assisted radical prostatectomy; RCT, randomized controlled trial; TP, transperitoneal.

## Supplementary Files

**Supplementary Table 1.** RCT Bias assessment and Cohort Study Quality using Newcastle – Ottawa Quality Assessment scale

**Supplementary Table 2.** Database Search Strategy
